# Supplementary material for: Coping strategies of women with obstetric fistula in the Northern Region of Ghana
Source: PLOS Ment Health. 2025 Sep 4;2(9):e0000418. doi: 10.1371/journal.pmen.0000418 (PMC12798545; doi:10.1371/journal.pmen.0000418)
Supplement: Attachment — Submitted filename: Response to Reviewers.docx [file pmen.0000418.s002.docx]

**Response to Reviewers**

**Reviewer 1**

2. Abstract

• Aim or objective should be separated from the background and appear as sub-section

The objective has been separated from the abstract.

• The key findings from this study are lacking in the results section

Key findings of the study have been clearly stated in the results section of the revised abstract

• Similarly the conclusion is not based on the findings of the study

The conclusion in the abstract has been revised based on the findings of the study

3. Introduction

• The term “Obstetric Fistula” need to be elaborated so that readers who are not health workers can understand what it means

The term ‘Obstetric Fistula’ has been explained in simple terms as ‘a condition where women leak urine or faeces without control due to a hole created between where babies are born through and where urine or faeces are stored. The condition leaves victims with body odour and unpleasant smells making it uncomfortable for others to come closer to them’.

4. Materials and methods section

• Line 84: should be Methodology instead of Materials and Methods

Materials and Methods has been changed to Methodology

• Line 89: The statement – It was the largest……; Has it stopped to be the largest?

Yes, the Northern Region is no longer the largest after the creation of the new regions. Savannah Region is now the largest region in Ghana.

• What was the selection criteria of the study participants considering both inclusion and exclusion criteria!!

The inclusion and exclusion criteria have been revised as;

Women diagnosed with obstetric fistula at the Fistula Centre, aged 18 years old or more and fluent in English, Dagbani or Twi languages were included. Women with obstetric fistula who did not understand English, Dagbani or Twi and those who could not be traced were excluded in the study

• How was this sample size of 15 women arrived at? And sample size seem to be small for the generalization of the findings

The sample size was arrived based on data saturation. There were no new themes by the 15^th^ respondent.

5. Results

• Table 1: Column of Duration of fistula should be “Duration of fistula (Months) and then remove the word month from the different patient values. And the duration of living with fistula by each participant, was it exactly whole numbers of months!!

The months have been removed from each of the participants’ duration with fistula but indicated in bracket in the heading ‘Duration of fistula’

• What were the outcomes of these coping mechanisms both positive and negative?

The positive outcomes of the coping strategies were prevention of constipation and reduction of odour due to incontinence.

The negative outcome of the coping strategies included skin rashes and itching as well as fear, anxiety and grief

6. Discussion

• What is the implication of the study findings?

The study findings provide insight for healthcare providers to intensify awareness on the availability of medical treatment for obstetric fistula. In addition, it is a call for policy direction to support treatment of obstetric fistula so as to facilitate access to medical treatment among women affected by the condition.

• Among the limitations mentioned, I think small sample size can affect the generalization of the findings

The findings of the study cannot be generalized due to the limited number of participants

7. Conclusion

• What were the main coping mechanisms that can be concluded from the findings?.

Women with obstetric fistula in the Northern Region of Ghana adopt two main coping strategies; problem-focused where the victims were found to take action toward finding a solution to their problems. They also adopt emotion-focused strategies where they received support from various sources toward finding a solution to their problem.

**Reviewer #2:**

- Overall, the methodology is robust, and the use of thematic content analysis ensures systematic and thorough data interpretation. However, providing additional details on the process for determining data saturation with 15 participants would improve the methodological rigor and strengthen the credibility of the findings.

As the data was collected, each transcript was constantly analyzed and new emerging themes were identified. This was repeated for each participant until new themes were no more emerging, thus signaling data saturation. In this study, data saturation was realized after the 15^th^ participant.

- Furthermore, expanding the discussion on cultural factors influencing coping strategies and healthcare-seeking behavior would provide a more nuanced interpretation of the results and deepen the reader’s understanding of the contextual challenges faced by the participants. The manuscript already mentions several cultural factors, such as stigma, traditional beliefs, and social expectations. However, expanding on how these cultural factors specifically influence the coping strategies of women with obstetric fistula and how they affect healthcare-seeking behavior would deepen the analysis.

Besides, socio-cultural factors including stigma and traditional beliefs serve as barriers for women to seek medical attention. In other words, the fears of what society would say about the condition do not give women the confidence to openly seek healthcare. Similarly, the strong traditional beliefs in Ghanaian societies where medical conditions are attributed to spiritual cause adversary impact the health seeking behavior of women with obstetric fistula.

- Although the study identifies various support systems such as family, healthcare workers, and neighbors, a more detailed analysis of the nature, extent, and adequacy of these support mechanisms could enhance the findings. In particular, elaborating on the role of healthcare providers in aiding coping strategies would add depth to the discussion.

In terms of family support, it was found that parents and siblings of participants offered them the moral support and cared for them before and during surgery. The also provided them with materials such as diapers and personal consumables as until the women treated and recovered from the condition.

- Additionally, while the authors have indicated that data can be obtained upon request, it is advisable to deposit anonymized data in a publicly accessible repository. This step would enhance transparency and reproducibility, aligning the manuscript with PLOS Mental Health’s data-sharing policies.

The study data is available at: doi **10.5281/zenodo.14918864**

**Reviewer #3:**

- Line 17 - colon and semi colon together after incontinence

The semi colon has been deleted

- Line 24 - Northern Region (More likely for readers to ask... of where?) Might be useful to emphasise that the area is called that or bracket Ghana.

Northern Region (Ghana)

- Also Line 507 - Capital first letters?

First letters changed to capital.

- Line 35 - Does this constitute body odour/ smells or anything physical that announces their condition? Useful to mention this as reader will need to know in lines 60-62 why employers won't keep them.

Due to the bad smell around them.

- Line 40 - space between as and being

Spaces between as and being has been removed

- Line 41, 56 and throughout - Check citation dates – APA
- Line 52 - please reword

Sentence reworded as,’ These can have a psychological, social and economic impact on women’

- Line 102 - Please reword - Were the women aged 18 years or more or diagnosed for 18 years or more? (confirmed in line 160 and Table 1)

Women aged 18 years old

- Line 130 - might be useful to summarise what the Colaizzi method is.

The Colaizzi method involves familiarization with data, identifying similar pattern, grouping common patterns into themes and providing in-depth description of themes.

- Line 152 - repeated phrase - years old; also, can assurance be given that the unknown age individual was not under 18 years old?

Yes, she affirmed to be older than 20 years ( Refer to transcript TF9)

- Line 156 - reword - four were farmers with one participant was...

..... with one participant being a farmer

- Line 157-missing comma

Comma introduced

- Line 172 - Table 1 - Itching and pains

Itching and pains

- Line 192 - Reconsider use of pampers except participants meant the specific brand - consider diapers or nappies?

Pampers have been replaced with diapers

- Line 329 - reconsider - take longer time

Longer time has been corrected to long time

- Line 520 - multiple, however.

Corrected effected

- Line 526 - "The PLOS Data policy requires authors to make all data underlying the findings described in their manuscript fully available without restriction, with rare exception."

Study data available at: doi **10.5281/zenodo.14918864**

Please add in dates with the citations, APA (Line 41) please revise reference citation to be as PLOS mental health guideline.

Ryan (2014)
