# Supplementary material for: Coping strategies of women with obstetric fistula in the Northern Region of Ghana
Source: PLOS Ment Health. 2025 Sep 4;2(9):e0000418. doi: 10.1371/journal.pmen.0000418 (PMC12798545; doi:10.1371/journal.pmen.0000418)
Supplement: Attachment — Submitted filename: Response_to_Reviewers_auresp_2.docx [file pmen.0000418.s003.docx]

**Dear Editor,**

**RESPONSE TO REVIEWERS**

Please, find our response to the reviewers comments as follows;

1. **Authors**

Author 2 and 3 institutional affiliation is not clear

Response

Institutional affiliation of 2 and 3 have been made clearer

2. **Abstract**

- Objective should to be “study assessed the Coping strategies of women with obstetric fistula in the Northern Region of Ghana

Response

**Objective:** The study assessed the Coping strategies of women with obstetric fistula in the Northern Region of Ghana.

- Conclusion is not based on the findings of the study and therefore it should be re-written.

Response

Conclusion has been re-written as, “Various coping strategies including use of chamber pots as seats, wearing of many clothes, use of rugs and pampers, avoiding bulky foods, frequent bathing and washing were employed by women with obstetric fistula to manage incontinence of urine and feces. In addition, the support of parents, siblings, health workers, neighbours and friends played a significant role in their daily lives. Despite these efforts, women with obstetric fistula experience physical and psychological effects include skin rashes, pain, fear and grief highlighting the adverse impact of obstetric fistula on the general well-being of women”

- What is this author summary? And it should be incorporated in the Introduction sub-section

Response

Author summary has been incorporated into the last paragraph of the introduction section.

3. **Introduction**

- Line 56-59: As authors try to define the term “Obstetric Fistula”; they should avoid such as “In simple words….

Response

“In simple words” has been removed from the definition of obstetric fistula

- Is there any literature available to support some of the coping mechanisms such women have developed?

Response

- Yes, Please refer to lines 86-88 in the manuscript

4. **Methodology**

• Line 114: The statement – It was the largest……; Has it stopped to be the largest?. This comment was raised but the authors have not tried to attempt to address it

Response

Yes, the Northern Region is no longer the largest after two new regions (Savannah and North East regions) were carved out of it. Savannah Region is now the largest region in Ghana.

• Line 127: Women who were not fluent in English, Dagbani, or Twi languages, they were excluded. And therefore if many of them were not fluent in those languages, they would not participate in the study. I don’t think this is enough to exclude them from the study thus creating selection bias. Let the author thinks through this exclusion criteria

Response

We have acknowledged selection bias as a limitation of our study since our exclusion criteria did not allow us to include women who could not express themselves well in English or Dagbani or Twi.

• Line 130: the statement “…traced were excluded in the study….”, how can one exclude what doesn’t exist?

Response

We have clarified the statement to read like “...those who could not be contacted after the interviews for follow-up were excluded in the study”.

• Who collected the data or administered the tool to the study participants?

Response

The first author did the data collection.

5. **Results**

 Table 1: what is the unit of age? Similarly, Duration of fistula (Months) should be “Duration lived with fistula (months)”

Response

The unit of age is “years” and duration of fistula (months) has been changed to “Duration lived with fistula (months)”

• In table 2: “Psychological effects of using incontinence products”, this statement is not clear. These individuals already experience incontinence, and so how can they go ahead and use incontinence products?

Response

“Psychological effects of using incontinence products” has been changed to “Psychological effects of coping strategies”

• These sub-themes in table 2, I suggest that they should be treatment separately especially those which are not similar. Otherwise the table is confusing on which is which?

Response

Please, the suggestion to treat sub-themes separately is not clear as how they should be separated. Kindly, indicate how we should do it and we will address that.

6. **Discussions**

• Line 522: The statement “Psychological side effects of coping strategies on women with obstetric fistula”, if these are coping mechanisms, how then can they cause psychological effects!!!

Response

“Psychological side effects of coping strategies on women with obstetric obstetric fistula” has been changed to “Psychological effects of coping strategies on women with obstetric fistula”

• Line 552: Limitations of the study, I think the small sample size can affect generalizability of the conclusion, any comment on this?

Response

The last sentence of the limitation has been revised to read “The findings of the study cannot be generalized due to the small sample size”

7. **Conclusion**
   • what were the main coping mechanisms that were found in the study such that a conclusion can be made on them? See the title and objectives of the study

Response

The main coping strategies were; problem-focused where the victims were found to take action toward finding a solution to their problems and emotion-focused strategies where they received support from various sources toward finding a solution to their problem.

**Reviewer #3:**

 This is very interesting and relevant piece of work. A brief definition of obstetric fistula can be added to the abstract to help readers understand the condition before reading further

Response

A brief definition of obstetric fistula has been added to the abstract
