# Supplementary material for: Coping strategies of women with obstetric fistula in the Northern Region of Ghana
Source: PLOS Ment Health. 2025 Sep 4;2(9):e0000418. doi: 10.1371/journal.pmen.0000418 (PMC12798545; doi:10.1371/journal.pmen.0000418)
Supplement: Attachment — Submitted filename: Response_to_Reviewers_auresp_3.docx [file pmen.0000418.s004.docx]

**Dear Editor,**

**RESPONSE TO REVIEWERS**

Please, find our response to the reviewers comments as follows;

Grammatical errors in Line 258, 323 and 557 and other errors identified have been corrected to provide clarity

1. **Abstract**
   Line 16-17: The statement “Participation was by written informed consent and interviews were recorded ……”is not clear and it needs to be paraphrased.

Response

Line 16-17 has been paraphrased to read ‘Consent of participants were sought, and interviews were recorded, transcribed verbatim, coded and……..’

2. **Introduction**

Line 38-42: The authors should merge the two sentences since they look similar. In line 49, the authors are mentioning that there many factors responsible for obstetric fistula, can the authors list them

Response

- Line 38-42 has been merged to read ‘Obstetric fistula is a condition in women where a hole is created between the birth canal and bladder and/or rectum due to prolonged or obstructed labour without access to timely medical treatment leading to uncontrollable leakage of urine and feces (1).
- Line 49 has been revised as ‘Many factors including lack of awareness, poor health seeking behaviour and lack of adequate health systems…….’

3. **Results**

The authors should have captured the associated risk factors to obstetric fistula by the study participants to cater for patient TF2. Patient TF2 in table 1, according to the definition given on obstetric fistula, the patient has no child and this assumes she has never given birth, can the authors explain why she had obstetric fistula? And this should be discussed in the discussion section.

Response

Please, in Table 1, participant TF2 has 2 children. Kindly refer to Table 1 in the manuscripts. All the participants except TF6 have children. According to TF6, she developed the fistula after the delivery of the first child who she lost. No participant therefore developed the condition due to other risk factors apart from delivery process

4. **Discussion**

The authors should discuss the associated risk factors to obstetric fistula and then cater for patient TF2

Response

Please, participant TF2 have children as captured in table 1
