# Supplementary material for: Coping strategies of women with obstetric fistula in the Northern Region of Ghana
Source: PLOS Ment Health. 2025 Sep 4;2(9):e0000418. doi: 10.1371/journal.pmen.0000418 (PMC12798545; doi:10.1371/journal.pmen.0000418)
Supplement: Attachment — Submitted filename: Response_to_Reviewers_auresp_4.docx [file pmen.0000418.s005.docx]

**Dear Editor,**

**RESPONSE TO REVIEWERS**

Please find our response to the reviewers’ comments as follows.

**Comment 1. Results**
The authors should address the issue of Patient TF6 in table 1, according to the definition given on obstetric fistula, the patient had no children, and this assumes she has never given birth, can the authors explain why she had obstetric fistula? And this should be discussed in the discussion section.

Response

We have taken notice of your concern about participant TF6. However, our results did not mean that the participant had fistula without history of childbirth. As a result, we have changed the heading ‘Number of children’ to ‘**Number of children alive’** in table 1 to make it clear. We hope this revision provides clarity to the childbirth status of participant TF6.

Thank you
